# Cause-related marketing: a systematic review of the literature

**DOI:** 10.1007/s12208-021-00326-y

**Published:** 2022-01-08

**Authors:** Hina Yaqub Bhatti, M. Mercedes Galan-Ladero, Clementina Galera-Casquet

**Affiliations:** 1 University of Extremadura, Badajoz, Spain; 2 Riphah International University, Lahore, Pakistan

**Keywords:** Cause-Related Marketing (CRM), Corporate Social Responsibility (CSR), Systematic Literature Review (SLR), Bibliometric Analysis, VOSViewer

## Abstract

Cause-Related Marketing (CRM) is one of the most versatile activities among the Corporate Social Responsibility (CSR) initiatives. Though CRM is extensively researched, however, only a few authors have performed systematic literature reviews on CRM. Therefore, more systematic reviews of CRM are still needed to complete and bring together the more contributions, advances, and different existing research lines. Thus, this paper provides a comprehensive overview of the existing literature in CRM from the two keywords: “Cause-Related Marketing” and “Cause Marketing”, and the time period ranges from 1988 to 2020. In this study, rigorous protocol is used in synthesizing 344 English articles drawing upon e-journal database searches. These articles were categorized by time-wise development, country-wise development, methodological development, cross-cultural analysis, the role of journals. This study also carried out the Bibliometric Analyses. The review highlights that the concept of CRM has evolved from being considered a marketing mix tool (a promotion tool), to being considered as a CSR initiative, with a more strategic character. Our findings revealed that only a few journals published articles on CRM. Geographically, the CRM study was initiated in North America, followed by Europe and Oceania, and Asian and Sub-Saharan African countries. From the third decade, there was more collaboration in cross-cultural studies and the use of mixed-method (qualitative and quantitative studies) approach. Lastly, this study shows the most manifest research gaps in CRM that opens avenue for future research.

## Introduction

Cause-Related Marketing (CRM) is a versatile and growing activity in the marketing field. It provides opportunities to profit and non-profit organizations, and consumers, to participate in a social cause (Varadarajan & Menon, 1988). Since 1988, CRM initiatives have gradually increased for more than three decades. CRM allows to achieve the societal and financial corporate establishment’s goals, as well as provide the opportunity to consumers to participate in an altruistic act.

**Fig. 1:**
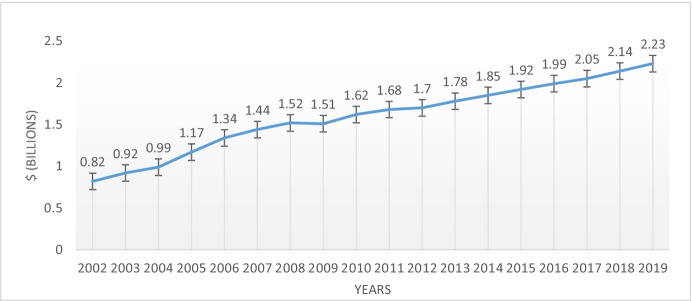
IEG Sponsorship Report from 2002 to 2019. Note: We only include data since 2002, because previous data are not available on the IEG Sponsorship Report. Source: IEG (2020)

Formally, the first CRM campaign named in this way was carried out by American Express (AMEX) in 1983, in the United States. The purpose of this program was to increase the usage of the AMEX credit card, but also collect money to be donated for the renovation of the Statue of Liberty. This project was developed from September to December, and the donation was $1.7 million (Varadarajan & Menon, 1988). Since then, and according to the IEG Sponsorship Report, cause sponsorship spending in North America has grown continuously^1^ from $120 million in 1990 until $2.23 billion in 2019, as shown in Fig. 1. In 2020, due to the COVID-19 pandemic, U.S. sponsorship value was $10 Billion (annually), which approximately increased 38% (IEG, 2020).

Consequently, the practice of CRM has also increased for the last three decades because more profit organizations have engaged in CRM activities (Adomaviciute et al., 2016), non-profit organizations have maintained environmental protection, health, and other worthy causes (Grolleau et al., 2016), and when consumers purchase the CRM products for support the cause, they have a prosocial behavior (Chang & Chu, 2020) and feel happy (Jeong & Kim, 2020; Vrontis et al., 2020).

During this time, CRM has become a topic of considerable debate in both managerial and academic circles worldwide. Although some systematic literature has been presented on this topic (see, for example, Guerreiro et al., 2016; Lafferty et al., 2016; Natarajan et al., 2016; or Thomas et al., 2020), an updated systematic literature review is required. Thus, we present a new systematic literature review: (1) To complete the review of the academic research articles in the area of CRM, from 1988 to 2020, with the perspective of profit organizations, non-profit organizations, and consumers over the last three decades; (2) To include cross- cultural studies; (3) To include studies carried out in developed and developing countries; (4) To include studies executed in different societies (e.g. Muslim societies, Western societies with Christian traditions, etc.); and (5) To conduct a bibliometric analysis using VOSViewer Software.

Thus, the main objective of this paper is to provide a systematic literature review of the existing research in the field of Cause-Related Marketing. More specifically, our aim is to find influential papers that have shaped this field and provide the overview of historical development in the field of research, focusing first on previously analyzed criteria: Time-Wise Development of CRM Literature, Country-Wise Development of CRM Literature, Methodological development in CRM Literature, and Role of Journal in Development of CRM literature. But also, this study carries out a systematic review with a bibliometric analysis. On the one hand, the systematic review helps the researchers to improve the rigor of prior reviewing literature. On the other hand, bibliometric analysis helps to analyze divergent views and examine the development of the CRM field.

Hence, this paper has followed two steps in the systematic literature review on CRM: (1) to select the inclusion and exclusion criteria, and (2) to analyze the evolution of CRM in seven different categories.

*First step*: Inclusion and Exclusion Criteria.

- This research only included published papers in journals, from 1988 to 2020 (data sources such as working papers, reports, newspapers, textbooks, conference papers, or theses / dissertations, were not included).
- Two keywords, “Cause-Related Marketing” and “Cause Marketing”, were used to search the databases (SAGE Publications, JSTOR, Emerald Full Text, Springer, John Wiley Publications, Elsevier, Taylor and Francis, and Google Scholar).
- This research also used conceptual review and empirical studies of different countries.
- This research only included papers written in English (i.e., non-English language research articles were excluded).
- This study considered the date of publication of the journal as the date of the research articles.

*Second step*: Academic researchers have used qualitative and quantitative methods for literature review to organize and provide the above underlying findings on CRM. And according to Liu et al., (2015), Bibliometric Analysis is a tool to examine literature review. Thus, this study has also provided a static and systematic picture of the research (Aria & Cuccurullo, 2017). This study relies on bibliometric techniques such as author-citation analysis, or co-words or co-occurrence analysis, and co-citation analysis of authors through VOSviewer software (version 1.6.5). Following Thomas et al. (2020), we selected Time-Wise Development of CRM Literature, Country-Wise Development of CRM Literature, and Methodological Development in CRM Literature. And we added other analysis such as Role of Journal in Development of CRM literature, author-citation analysis, and Co-words or Co-occurrence analysis, proposed by Poje & Groff (2021). We also considered adding a new category that had not been considered in previous studies: cross-cultural analysis.

Therefore, the structure of this paper is organized as follow: firstly, we show the CRM theoretical background (with a previous introduction to CSR, to link it with CRM, because CRM is generally considered under the umbrella of CSR – e.g., Kotler and Lee, 2005; Galan-Ladero, 2012); secondly, we offer the results of our literature analysis in the CRM field; later, we discuss these results; and finally, we offer the main conclusions, also considering the main limitations of this study and further research.

## Background

Since the inauguration of the third millennium, Corporate Social Responsibility (CSR) has become a globally hot issue by the rapid change of the environment. A large number of organizations, from developed and developing countries, have focused on CSR.

CSR, defined as *“a concept whereby companies integrate social and environmental concerns in their business operations and in their interaction with their stakeholders on a voluntary basis*” (European Commission, 2001), has a wide range of history: it started in Western countries, but later, it spread all over the world. Antecedents of CSR can be found at 18th and 19th Centuries, with the creation of welfare schemes adopted with a paternalistic approach, to protect companies and retain employees with improved life quality (Carroll 2008). But it is in the twentieth Century, and specifically after World War II, when scholars and practitioners discussed about the social responsibilities, and successful businesses also adopted such responsibilities (Heald, 1970).

Thus, CSR started to be established and, in the last seven decades, it has played different roles:

- ***The 1950s*** was the first era that established the current CSR. Successful business leaders and board of directors moved towards the ethics of society. Bowen, the first who coined the term, introduced the concept, and provided the initial definition of CSR, described as “*the obligations of businessmen to pursue those policies, to make those decisions, or to follow those lines of action which are desirable the objectives and values of our society*” (Bowen, 1953, p. 6). In this area, Heald (1957), discussed that businesses do not only serve on economic performance work, but they also serve on humane and constructive social policies.
- ***The 1960s***: Many of the definitions of CSR are formalized. Walton (1967) was a prime thinker who addresses the different aspects of CSR: “*In short, the new concept of social responsibility recognizes the intimacy of the relationships between the corporation and society and realizes that such relationships must be kept in mind by top managers as the corporation and the related groups pursue their respective goals (Walton,* 1967*, p. 18)”.*
- ***The 1970s***: Friedman described that the social responsibility of business is to enhance profits and maximize shareholder value. Therefore, Carroll (1979) came in this decade with the new concept of CSR, defined as “*the social responsibility of business encompasses the economic, legal, ethical, and discretionary expectations that society has of organizations at a given point in time*”.
- ***The 1980s***: the notions of stakeholder management and business ethics had become the main integral part of the business (Carroll, 2008). In 1980, Jones proposed that CSR is a process, not the outcome, and CSR, when engaged in as a process of decision making, should constitute CSR behavior by the corporation (Jones, 1980). Also, Aupperle et al., (1983) suggested that four aspects include CSR: economic, legal, ethical, and voluntary or philanthropic responsibilities.
- ***The 1990s***: Carroll (1991) revised the concept of CSR and introduced the “Pyramid of Corporate Social Responsibility”. He described four main responsibilities of the company: economic responsibility (“be profitable”), legal responsibility (“obey the laws and regulations”), ethical responsibility (“do what is just and fair”), and philanthropic responsibility (“be a corporate citizen”). During this decade, Elkington (2001) introduced another concept of CSR, the “Triple Bottom Line”, which focuses on three issues: social responsibility (“people”), environmental responsibility (“planet”), and economic responsibility (“profit”).
- ***In the first decade of the twenty-first century (The 2000s),*** CSR extends to emerging markets. After the collapse of Enron,^2^ many organizations and corporations focused on establishing CSR departments, hiring CSR consultants and CSR managers. On the other hand, in 2002, ISO Committee on Consumer Policy play an important role in ISO 26000, an international standard that present a guideline on Corporate Social Responsibility.^3^
- ***In the second decade of the twenty-first century (The 2010s),*** Kramer and Porter (2011) introduced the concept of “creating shared value”, which becomes the core objective of business strategies. 2015 is an important year because the “2030 Agenda for Sustainable Development”, with the “Sustainable Development Goals” (SDGs), was launched. SDGs covered a wide range of global areas, such as fighting against climate change, removing poverty and hunger, as well as promoting sustainable consumption, among others.

Therefore, different theories have been created and adapted during all this time. The most important theories are Carroll’s CSR Pyramid Theory,^4^ Triple Bottom Line Theory,^5^ Stakeholder Theory,^6^ and Corporate Citizenship Theory.^7^

On the other hand, CSR initiatives, formed as a part of the core business activities, provide long-term financial security and growth for stakeholders but also increase the market position (Bhattacharyya et al., 2008). Under the big umbrella of CSR, different initiatives have appeared, and they have become growing popular among profit organizations worldwide. Kotler et al., (2012) explained six different types of CSR initiatives (see Table 1), which included cause promotion, cause-related marketing, corporate social marketing, corporate philanthropy, community volunteering, and socially responsible business practices.

**Table 1:** Main CSR Initiatives

Corporate Social Marketing	Cause-Related Marketing	Cause Promotion	Corporate Philanthropy	Socially Responsible Business Practices	Community Volunteering
Supporting behavior change campaigns	Making a contribution or donating a percentage of revenues to a specific cause based on the product sales or usage	Supporting social causes through promotional sponsorships	Making direct contributions to a charity or cause	Adapting and conducting discretionary business practices and investments that support social cause	Supporting employees to volunteer in the community

Source: Adapted from Kotler and Lee (2005), and Kotler et al. (2012)

According to Thomas et al., (2011), CSR has received significant attention in both academic and business societies. CRM, as one of these initiatives, has progressed in social responsibility and allows firms to link their philanthropic activities and strategic marketing goals. On the other hand, CRM activities also have been an increasing part of the corporate marketing plans (Gupta & Pirsch, 2006a). Therefore, this study especially focuses on this CSR initiative: Cause-Related Marketing (CRM).

The first definition of Cause-Related Marketing (CRM) was introduced by Varadarajan and Menon (1988, p. 60), as “*the process of formulating and implementing marketing activities that are characterized by an offer from the firm to contribute a specified amount to a designated cause when consumers engage in revenue-providing exchanges that satisfy organizational and individual objectives”.* This definition provides two main streams: to support the charitable cause and to satisfy organizational and individual objectives.

On the other hand, the most essential and significant benefit of the CRM is shown as a win-win-win situation (for the profit organizations, non-profit organizations, and consumers - Adkins, 1999). CRM campaigns increase the number of sales for the organization, as well as enhance the number of donations to the non-profit organizations (Deb & Amawate, 2019). CRM campaigns also give the best chance to profit organizations to attract the customers towards organization and enhance customer loyalty (Galan-Ladero et al., 2013b), as well as they create or enhance emotional engagement with target customers, build a strong relationship with them (Cone et al., 2003; Docherty & Hibbert, 2003), and also maintain the company’s goodwill (Chang & Chu, 2020). Consequently:

- ***The for-profit organizations*** use CRM as a strategic tool to build a strong brand image in the customer’s mind (Ahluwalia & Bedi, 2015). And the internal benefit of the for-profit organization is to help increase the employee’s self-esteem, commitment, and loyalty (Cone et al., 2003; Polonsky & Wood, 2001).
- ***The non-profit organizations*** try to increase awareness about the cause, educate the customers, and support the charitable cause (Nowak and Clarke, 2003). On the other hand, CRM in non-profit organizations increases the number of donors (Docherty & Hibbert, 2003; Polonsky & Wood, 2001).
- ***For consumers***, charitable causes, linked to their purchases, boost their feeling of happiness and inner satisfaction (Chaabane & Parguel, 2016), and they also feel good when helping others (Imas, 2014

- 2014).

## Analysis and Main results

Due to an increasing number of CRM research papers that identify the most essential and main contributions in the field, and to objectify the outcomes, then bibliometric analysis is introduced. Zupic and Čater (2015) explained the five main bibliographic methods, which consists of citation analysis, co-citation analysis, bibliographic coupling, co-author analysis and co-word analysis. In this study, we apply Co-words or Co-occurrence analysis, Co-citation analysis, and cited journals analysis. These analyses were run on VOS-software.

### Analysis of the different definitions of CRM

A wide variety of definitions of CRM have been contributed since 1988 (see Appendix 1, Table 7). In Table 2, we summarize the main CRM definitions, from 1988 to 2020, according to the main keywords included in them: CRM as an activity (a marketing activity and/or a CSR activity), as a strategy, as a marketing mix tool, and as an alliance (between profit and nonprofit organizations). Thus, we can observe that there is not a general, unanimous agreement about its definition yet. However, the concept of CRM has evolved from being considered a short-term marketing mix tool (a promotion tool), to being considered a CSR initiative, with a more strategic character.

**Table 2:** Main Keywords in CRM definitions

**Keywords**	**Authors**
CRM is an activity	Varadarajan and Menon (1988) Hawkens and Stead (1996) Mullen (1997) Adkins (2007) Pringle and Thompson (1999) Hajjat (2003) Kotler and Lee (2005) Van den Brink et al. (2006) Gupta and Pirsch (2006b) Larson et al. (2008) Galan-Ladero (2012) Sabri (2018)
CRM as a strategy	Smith and Alcorn (1991) Barone et al., (2000) Endacott (2004) Fromherz (2006) Thamaraiselvan et al., 2017 Jung et al., 2018 Manojkumar and Sharma (2018) Yun et al., 2019) Srivastava (2020)
CRM as a marketing mix tool	File and Prince (1998) Kim and Lee (2009) Tangari et al. (2010) Beise-Zee (2013) Boenigk and Schuchardt (2013) Stumpf and Teufl (2014) Pringle and Thompson (2001) Samu and Wymer Jr (2001) Bergkvist and Taylor (2016)
CRM as an alliance (between profit and nonprofit organizations)	Carringer (1994) Ptacek and Salazar (1997) Webb and Mohr (1998) Nowak and Clarke (2003) Docherty and Hibbert (2003) Cui et al. (2003) Berglind and Nakata (2005) Lafferty and Goldsmith (2005) Chéron et al. (2012)

Source: Own Elaboration, based on Galan-Ladero et al. (2013a, b)

### Time-wise development of CRM literature

However, in this study, we start from the time-wise development of Cause-Related Marketing. First, we identify the number of research articles into three time periods (decades): 1988–2000, 2001–2010, and 2011–2020 (previous systematic literature did not classify them into decades). With the growing body of Cause-Related Marketing, it is better to organize it in three decades because differences are appreciated, depending on the time.

Varadarajan and Menon introduced the CRM term in academia in 1988, and the following three decades witnessed gradual growth in CRM literature. Table 3 shows the annual evolution of this figure from 1988 to 2020.

**Table 3:** The list of Time-Wise Development of CRM Literature

1988–2000			2001–2010			2011–2020		
Year	Absolute Frequency	Accumulated Absolute Frequency	Year	Absolute Frequency	Accumulated Absolute Frequency	Year	Absolute Frequency	Accumulated Absolute Frequency
1988	1	1						
1991	2	3	2001	6	6	2011	10	10
1992	1	4	2002	3	9	2012	18	28
1993	1	5	2003	13	22	2013	24	52
1994	–	–	2004	4	26	2014	26	78
1995	1	6	2005	4	30	2015	14	92
1996	–	–	2006	6	36	2016	19	111
1997	–	–	2007	9	45	2017	12	123
1998	3	9	2008	8	53	2018	16	139
1999	1	10	2009	10	63	2019	35	174
2000	3	**13**	2010	11	**74**	2020	83	**257**

Source: Own Elaboration

Thus, we can classify the three decades based on CRM literature progression:

i. Introductory decade (1988–2000). The field of CRM was introduced in this period with a limited number of published articles (13). However, these articles were very innovative and aroused interest in this new solidarity initiative.
ii. Emerging decade (2001–2010). The CRM field grabbed the attention of researchers in this second decade, with a notable increase in the published literature, especially in the last two years of this decade. The number of published articles reached 74. Consequently, CRM became an interesting and novel research topic, broadening its scope.
iii. Most thriving decade (2011–2020). CRM literature witnessed a boom in the third decade, especially in the last two years of this decade. Thus, 257 articles related to the field of CRM were published in different journals only in this third decade.

In summary, we can indicate that CRM publications have grown significantly over the three decades analyzed, because more and more research papers have been published on this topic.

### Author-based citation analysis

Author-based studies have long been one of the most important aspects of bibliometric analysis. This analysis includes the ranking of authors by the number of researches carried out, the citations of their research articles, their evolutions, or the analysis of co-authors’ collaborations. Table 4 shows the five most cited authors (and their specific works) from first decade (1988–2000), second decade (2001–2010), and third decade (2011–2020).

**Table 4:** The list of five most cited authors (and their specific works) from each decade

1988–2000		2001–2010		2011–2020	
Authors	Citations	Authors	Citations	Authors	Citations
Varadarajan (1988)	734	Barone (2007)	243	Christofi (2020a)	176
Webb (1998)	498	Gupta (2006a)	189	Robinson (2012)	150
Smith (1991)	121	Lafferty (2005)	187	Bae (2017)	149
File (1998)	110	Cui (2003)	152	Priporas (2020)	135
Ross (1992)	15	Berglind (2005)	103	Koschate-fischer (2012)	129

Source: Own Elaboration

In this analysis, the most cited authors (and their corresponding works) for each decade have been the following:

- From the first decade, the most cited authors are: Varadarajan (1988), with 734 citations; followed by Webb (1998), with 498; Smith (1991), with 121; File (1998), with 110; and Ross (1992), with 15 citations.
- In the second decade, Barone (2007) is the most quoted, with 243 citations; followed by Gupta (2006a), with 189; Lafferty (2005), with 187; Cui (2003), with 152; and Berglind (2005), with 103 citations.
- During the third decade, Christofi (2020a) has been cited 176 times; Robinson (2012) has 150; Bae (2020), 149; Priporas (2020), 135; and Koschate-Fischer (2012), 129 citations.

In summary, we can highlight that Varadarajan (1988) is the most cited author of all time, with the first academic paper published on CRM, and serves as a reference for researchers around the world. And by far the next most cited authors are Webb (1998) and Barone (2007).

### Co-words or co-occurrence analysis

A co-word analysis may be described as “*a content analysis technique that uses patterns of co-occurrence of pairs of items… in a corpus of texts to identify the relationships between ideas within the subject areas*” (He, 1999, p. 134). Thus, co-words or co-occurrence analysis is a content analysis that connects words in the title of the research paper or abstract. The main idea of the co-word analysis is to connect any identified patterns into a map of contextual space. We also applied this analysis to each decade.

#### First phase (period 1988–2000)

For the 13 articles published from 1988 to 2000, the co-word analysis identifies four clusters consisting of the following words (with the minimum number of occurrences of keywords defined as 1; out of 26 keywords in this period, 26 met the threshold).

**Fig. 2:**
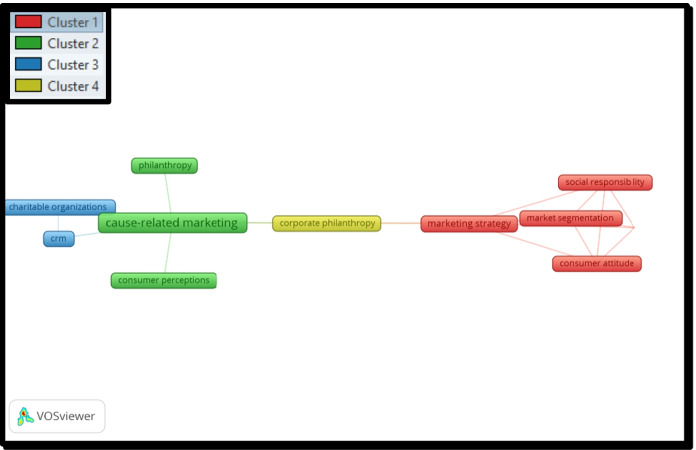
Co-Word analysis for the period 1988 to 2000

- The first cluster includes *consumer attitude, market segmentation, marketing strategy, profitability,* and *social responsibility* (as shown in Fig. 2, red color).
- The second cluster deals with *cause-related marketing, consumer perceptions,* and *philanthropy* (as shown in Fig. 2, green color).
- The third cluster consists of *charitable organizations* and *crm* (as shown in Fig. 2, blue color).
- The last cluster relates to *corporate philanthropy* (as shown in Fig. 2, yellow color).

In summary, the co-word analysis shows that, for the period from 1988 to 2000, the focused keyword is *Cause-Related Marketing*. Other important keywords are *philanthropy* and *consumer perception*.

#### Second phase (period 2001–2010)

Based on the selection of 74 articles for the period 2001–2010, the co-word analysis shows a more precise picture than it does in the introductory decade (to narrow down the result, the minimum number of the occurrence of keywords was defined as 2; out of 160 keywords, and 27 meet the reduction criteria).

**Fig. 3:**
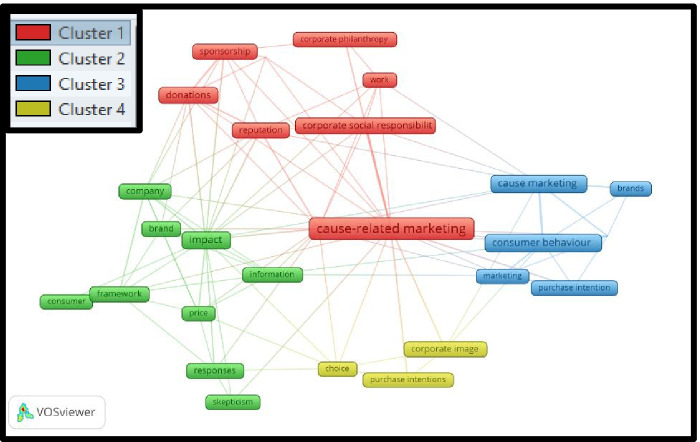
Co-word analysis for the period 2001 to 2010

- A notable cluster derived by the co-word analysis (Fig. 3, red color) consists of the words *brand alliances, cause-related marketing, corporate philanthropy, corporate social responsibility, donations, reputation, social responsibility, sponsorship,* and *work*.
- A second cluster (Fig. 3, green color) comprises *brand, company, consumer, framework, impact, information, price, responses,* and *skepticism.*
- The third cluster (Fig. 3, blue color) is related to *advertising, brand, cause marketing, consumer behavior, marketing,* and *purchase intention*.
- The fourth cluster (Fig. 3, yellow color) consists of *choice, corporate images,* and *purchase intention.*

In summary, the co-word analysis shows that, from 2001 to 2010, the *Cause-Related Marketing* keyword related to the other striking keywords, such as *corporate social responsibility, reputation, corporate image*, and *purchase intention*.

#### Third phase (period 2011–2020)

**Fig. 4:**
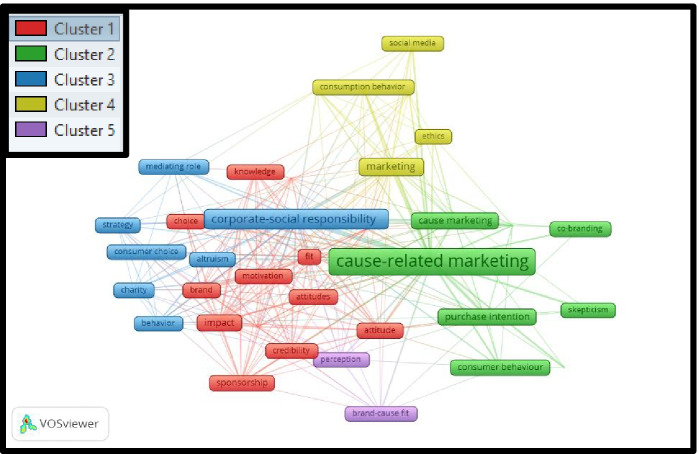
Co-word analysis for the period 2011 to 2020
- The second cluster (Fig. 4, green color) comprises *altruism, cause marketing, cause-related marketing, co-branding, consumer behavior, purchase intention, and skepticism.*
- The third cluster (Fig. 4, blue color) comprises *altruism,* *behavior,* *charity,* *consumer choice,* *corporate social responsibility,* *mediating role,* and *strategy*.
- The fourth cluster (Fig. 4, yellow color) comprises *consumption behavior, corporate strategy, ethics, marketing, millennials,* and *social media.*
- The fifth cluster (Fig. 4, purple color) comprises *brand-cause fit, corporate social responsibility,* and *perceptio*n.

Based on the selection of 257 articles for the period 2011–2020, the co-word analysis shows a more precise picture than it does in the previous two decades. To narrow down the result, the minimum number of the occurrence of keywords was defined as 5 (out of 825 keywords, and 40 meet the reduction criteria). The most notable clusters derived from the co-word analysis are:

- First cluster (Fig. 4, red color): it consists of the words *attitude, attitudes, brand, choice, consumer responses, credibility, fit, impact, knowledge, motivation, responses, social-responsibility, sponsorship,* and *sustainability.*

In summary, the co-word analysis shows that, also for the period from 2011 to 2020, the most focused keyword is *Cause-Related Marketing*. Other significant keywords are *ethics*, *purchase intention*, *consumer behavior*, and *attitudes*.

Consequently, the whole co-word analysis shows that all around the world, the researchers are focused on the one keyword that is “*Cause-Related Marketing*”, and the other most emphasis keywords are *philanthropy* and *consumer perception*, in the first decade; to evolve toward *CSR, reputation, corporate image*, and *purchase intention*, in the second decade; and finally focused on *ethics, purchase intention, consumer behavior*, and *attitudes*, in the third decade.

### Co-citation analysis

A co-citation analysis is described as “*the scholarly contributions of authors who are frequently co-cited are likely to embody similar or related concepts*” (Nerur et al., 2008, p. 321). Co-citation analysis can explain how referential frameworks of the Cause-Related Marketing field at different stages of its development affected evolutions in its general construction.

#### First phase for the period 1988–2000

**Fig. 5:**
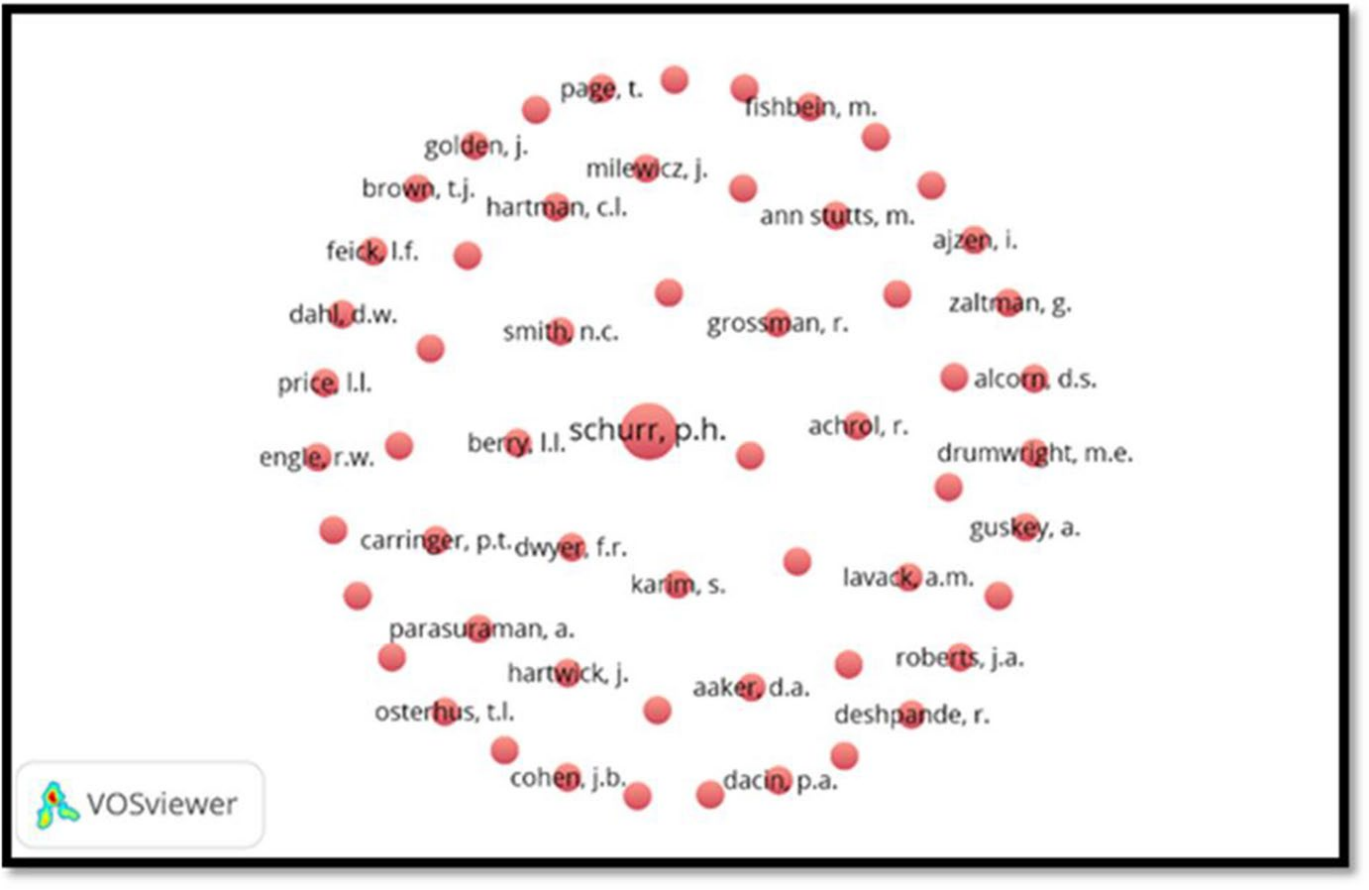
Co-citation analysis of authors for the period 1988–2000

Based on the co-citation analysis, for the period 1988-2000 (Fig. 5), it has been seen that there is predominately one cluster with a total 58 authors distributed in one cluster namely cluster – 1 with red color (minimum of the documents for an author should 1 and minimum citation of an author should be 1).

There is a wide variety of authors cited in the papers on CRM in the first decade (but only once). *Schurr* is the only author who receives 2 citations in this decade.

#### Second phase for the period 2001–2010

**Fig. 6:**
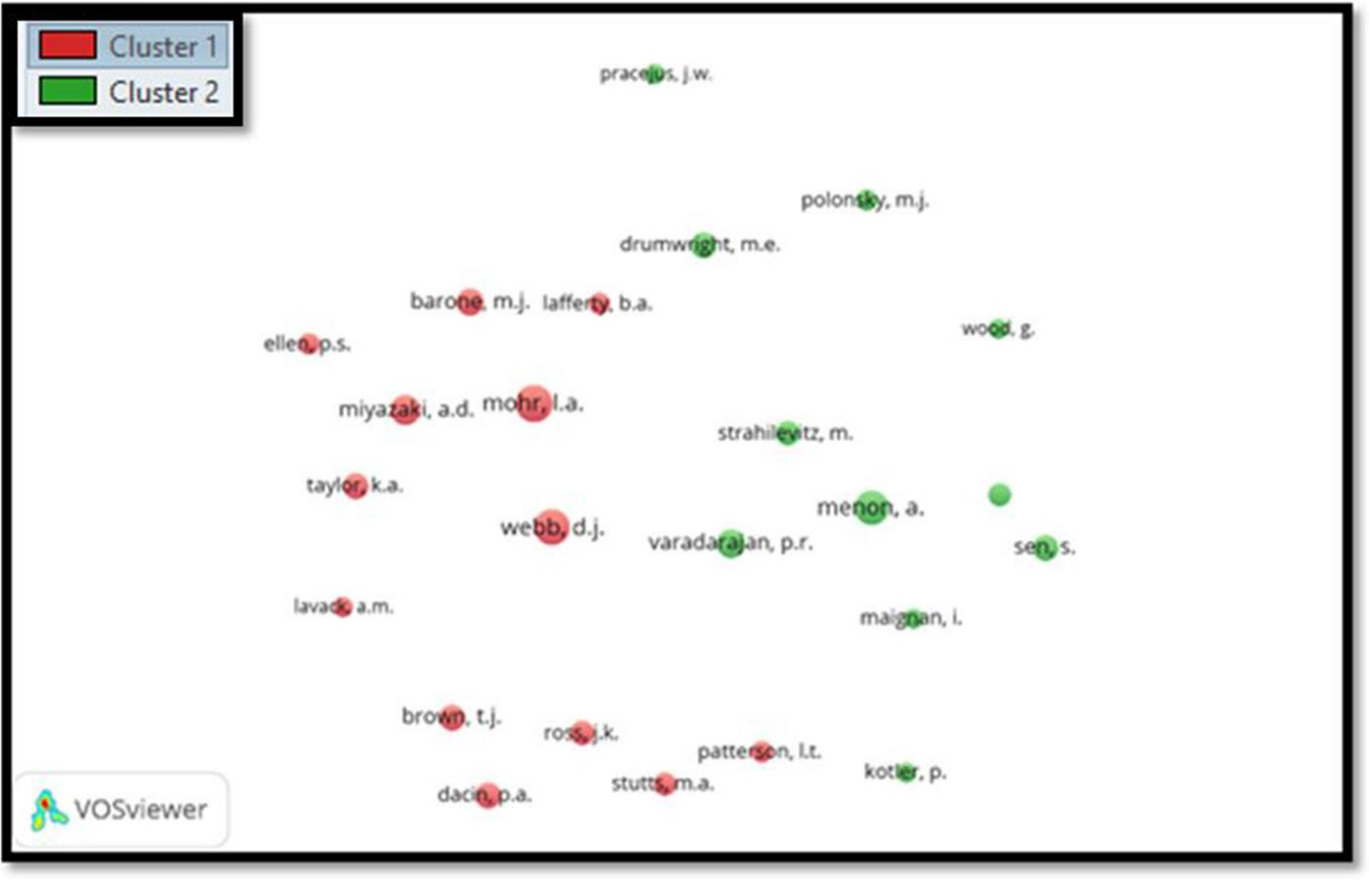
Co-citation analysis of authors for the period 2001–2010

According to co-citation analysis for the period 2001-2010 (Fig. 6), it has been noted that there are predominately two clusters with a total 1502 authors (minimum citation of an author should be 10 and the maximum citation of the author should be 24).

In this analysis, we selected five top co-authors who have a high citation, such as Mohr (34 citations), Webb (32 citations), Menon (30 citations), Miyazaki (24 citations), and Varadarajan (21 citations).

#### Third phase for the period 2011–2020

**Fig. 7:**
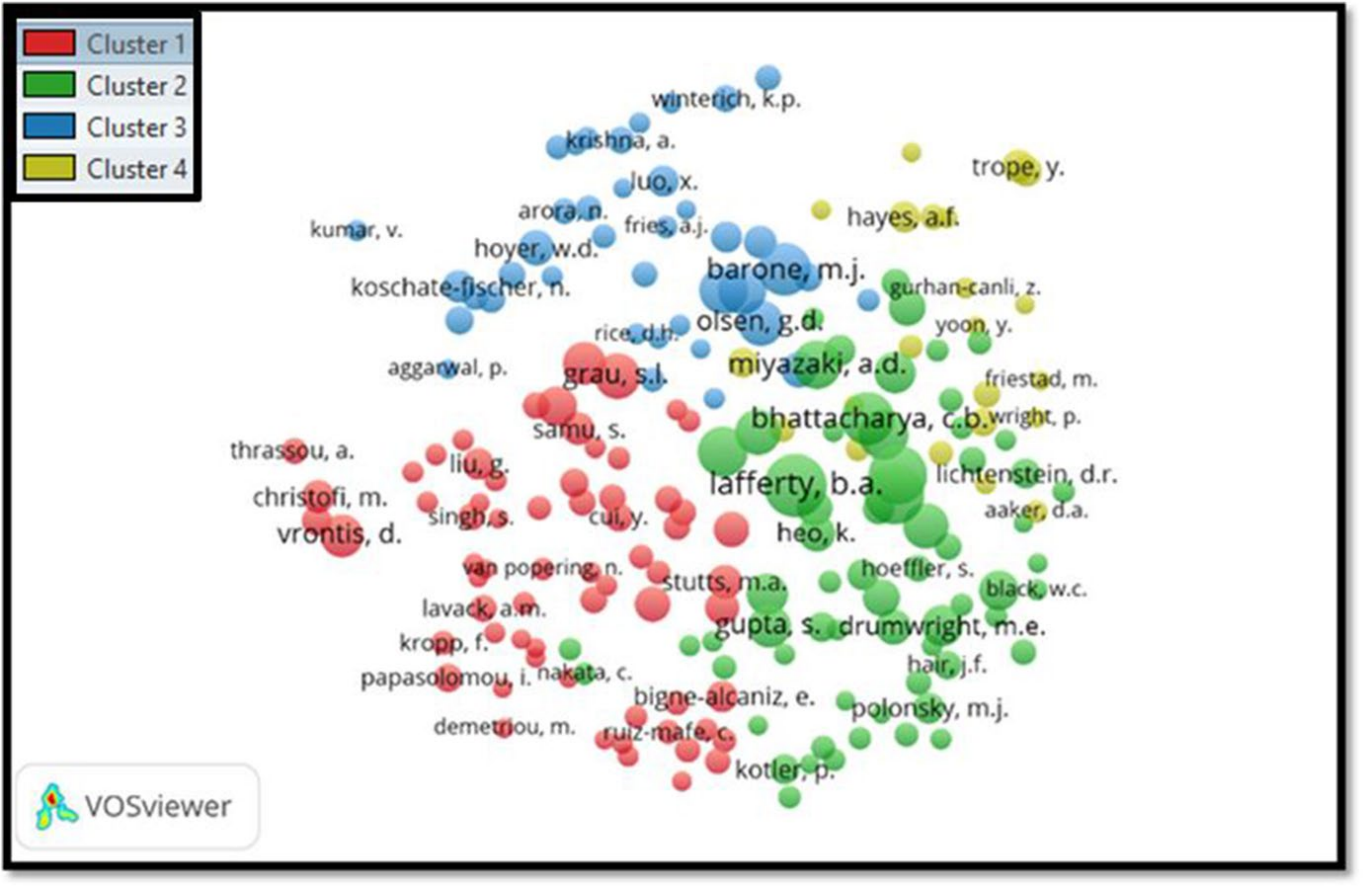
Co-citation analysis of authors for the period 2011–2020

According to co-citation analysis for the period 2011-2020 (Fig. 7), it has been noted that there are predominately four clusters, with a total of 10,108 authors and 193 thresholds (minimum of the documents for an author should be 20 and minimum citation of an author should be 5; the maximum citation of the author should be 200).

In this analysis, we selected the five top co-authors who had the highest citation, such as Lafferty (200 citations), Mohr (176 citations), Webb (167 citations), Barone (136 citations), and Bhattacharya (136 citations).

In summary, the co-citation analysis shows that Lafferty is the most co-cited author in all time. And the next most co-cited authors are Mohr and Webb.

### Cross-cultural analysis

Cultural norms and beliefs have a significant impact on shaping people’s perceptions, attitudes, and behavior (Steenkamp, 2001). Lavack and Kropp (2003) identified the research gap of cross-cultural studies in the field of CRM. Hence, they conducted the first cross-cultural research in the field of CRM by including four countries from different regions such as Australia (Oceania), Canada (North America), Korea (East Asia) and Norway (Europe), and investigated the consumers’ role values towards the CRM. Since the third decade, more researchers have been participating and collaborating in cross-cultural studies. Table 5 details transversal research that has studied CRM comparing different countries.

**Table 5:** Cross-Culture Analysis

Author/s (Year)	Countries	Article Titles
Cosgrave and O’Dwyer (2020)	Ireland and United Arab Emirates	Ethical standards and perceptions of CRM among millennial consumers
Bautista Jr. et al., (2020)	USA and d Philippines	Will cause-related marketing affect the American and Filipino college students’ purchase intention?
Pandey et al. (2020)	India and Philippines	An Experimental Approach to Examine the Antecedents of Attitude, Intention, and Loyalty Towards Cause-related Marketing: The Case of India and the Philippines.
Schyvinck and Willem (2019)	Belgium, Netherlands, France and United Kingdom	From cause-related marketing strategy to implementation in professional basketball organizations: a matter of alignment.
Santoro et al., (2019)	Italy and Japan	Cause-related marketing, brand loyalty and corporate social responsibility: A cross-country analysis of Italian and Japanese consumers
Woo et al., (2019)	USA and South Korea	Is this for our sake or their sake? Cross-cultural effects of message focus in cause-related marketing
Ferraris et al., (2019)	Italy and Brazil	Refining the relation between cause-related marketing and consumers purchase intentions.
Heidarian (2019)	Iran and Germany	The impact of trust propensity on consumers’ cause-related marketing purchase intentions and the moderating role of culture and gender
Bae (2017)	USA and South Korea	Matching cause-related marketing campaign to culture
Hawkins (2015)	India and USA	Shifting conceptualizations of ethical consumption: Cause-related marketing in India and the USA
Wang (2014)	China and USA	Individualism/collectivism, charitable giving, and cause-related marketing: a comparison of Chinese and Americans
Vaidyanathan et al., (2013)	USA and Poland	Interdependent self-construal in collectivist cultures: Effects on compliance in a cause-related marketing context
La Ferle et al., (2013)	USA and India	Factors impacting responses to cause-related marketing in India and the United States: Novelty, altruistic motives, and company origin
Kim and Johnson (2013)	USA and South Korea	The impact of moral emotions on cause-related marketing campaigns: A cross-cultural examination
Lavack and Kropp (2003)	Canada, Australia, Norway and South Korea	A cross-cultural comparison of consumer attitudes toward cause-related marketing

Source: Own Elaboration

According to the cross-sectional analysis, the nation has a different background of consumer and corporate cultures that varies from country to country. Sekaran (1983, p. 68) defined it as “*Culturally normed behavior and patterns of socialization could often stem from a mix of religious beliefs, economic and political exigencies and so on. Sorting these out in a clear-cut fashion would be extremely difficult, if not totally impossible*”. Therefore, the scholars are taking more consideration in cross-cultural CRM study from the second decade. In this study, Table 5 shows that researchers from USA (i.e., North America) and South Korea (i.e., East Asia) studied together two times on culture analysis, one times with India (i.e., South Asia), and one time with Poland (i.e., Europe), one times with Philippines (i.e., East Asia) as well as China (i.e., East Asia). In addition, Italian researchers (i.e., Europe) studied one time on culture analysis with Japan (i.e., East Asia) and one time with Brazil (i.e., South America). Furthermore, India (i.e., South Asia) collaborated with Philippines (i.e., East Asia). Cross-cultural analysis in Table 5 shows that the researchers worked on four different cultures analysis rather than two cultures (i.e., Lavack & Kropp, 2003; and Schyvinck & Willem, 2019).

### Country-wise development of CRM literature

**Fig. 8:**
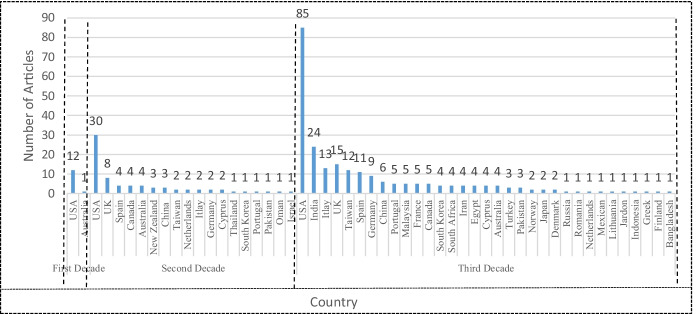
List of cited Country-Wise Development of CRM Literature. Source: Own Elaboration

Figure 8 reports regional (i.e., country-wise) participation of different researchers in the development of CRM literature. As this concept was introduced in the USA (Varadarajan and Menon, 1988), the studies from the first and second decades usually belonged to this geographical region. Thus, most of the research in CRM literature was published by researchers from US Universities: 92% of the contributions in the first decade (i.e., 12 research articles), and 40% (i.e., 30 research articles) in the second decade. However, some British researchers also contributed to CRM literature in the second decade, with 11% (i.e., 8 research articles) share of total CRM publications. Nevertheless, in the third decade, most of the CRM literature was published by Asian researchers. Hence, Indian researchers, with 9% (i.e., 24 research articles), and Taiwanese scholars, with 5% (i.e., 12 research articles), jointly published almost 14% of the articles in that decade. Although the American contributions fell to 33% (but only in relative terms, since in absolute terms they reached 85 research articles), their overall contribution remains the highest of all countries. And participation of British scholars was 6% (i.e., 15 research articles) in the third decade. In this Fig. 8, we observed that the USA research publications from every decade are very extensive, in comparison to other countries.

### Methodological development in CRM literature

**Fig. 9:**
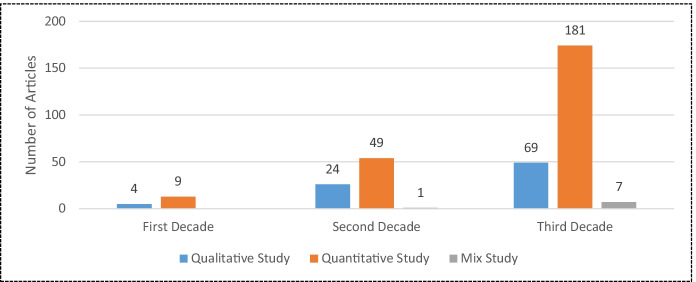
Methodological development in CRM Literature. Source: Own Elaboration

Methodological development in CRM literature is graphically shown in Fig. 9. It is observed that most research work is employed by experimental design. In the first decade (1988–2000), researchers focused on qualitative or quantitative research in the field of CRM; whereas a mixed-method approach has been used in the second (2001–2010) and third decade (2011–2020). In this analysis, we observe that, in general, quantitative studies significantly outnumber qualitative studies, especially in the third decade.

### Role of journal in development of CRM literature

Although a total of 141 journals have published articles explaining the concept of CRM from different perspectives (see Appendix 2, Table 8), only six journals published more than ten CRM papers. These journals are *International Marketing Review* (26), *International Review on Public and Nonprofit Marketing* (20), *Journal of Nonprofit & Public Sector Marketing* (17), *International Journal of Nonprofit and Voluntary Sector Marketing* (15), *Journal of Business Ethics* (13), and *Journal of Business Research* (10). In the field of CRM, almost 100 journals have been published only a single article since its conceptualization in 1988 (they are also shown in Appendix 2, Table 8).

There are two indicators to measure the scientific influence of scholarly journals, such as Journal Citation Reports and Scimago Journal & Country Rank. This study only considers the Scimago Journal & Country Rank because there are more research articles in this rank, which divides the set of journals into four quartiles (i.e., Q1, Q2, Q3, and Q4). According to Scimago Journal & Country Rank (SJR), we observed that 51 journals publishing about CRM are Q1, 36 are Q2, 16 are Q3, and 5 are Q4. On the other hand, 8 journals are not included yet in any Quartil. and 25 journals are not in this index.

According to Persson et al. (2009), for the bibliographical data, we used BibExcel, which presents co-occurrence of references in the bibliographic of research articles. Therefore, in this study we find that five most cited journals by each decade, from the first decade (1988–2000), are: *Journal of Marketing* (1 document; cited in 734 articles), *Journal of Public Policy & Marketing* (1 document; cited in 498 articles), *Journal of Consumer Marketing* (1 document; cited in 121 articles), *Journal of Business Ethics* (1 document; cited in 110 articles), and *Journal of the Academy of Marketing Science* (2 document; cited in 51 articles). From the second decade (2001–2010): *Journal of Consumer Marketing* (3 documents; cited in 369 articles), *Journal of Business Research* (3 documents; cited in 280 articles), *Journal of Retailing* (1 document; cited in 243 articles), *Journal of Nonprofit and Public Sector Marketing* (11 documents; cited in 215 articles), and *Journal of Advertising* (5 documents; cited in 172 articles). And from the third decade (2011–2020): *Journal of Marketing Review* (25 documents; cited in 842 articles), *Journal of Business Ethics* (11 documents; cited in 458 articles), *International Journal of Nonprofit and Voluntary Sector Marketing* (9 documents; cited in 407 articles), *International Journal of Advertising* (8 documents; cited in 347 articles), and *Journal of Marketing* (2 documents; cited in 244 articles).

For this analysis, we observed that the percentage of the most cited paper, published in the *Journal of Marketing*, dropped due to the introduction of different journals, such as *Journal of Marketing Review*, *International Journal of Advertising*, or *International Journal of Nonprofit and Voluntary Sector Marketing*. But, on the other hand, the *Journal of Business Ethics* has increased the citations.

## Discussion

This research provides an inclusive review of the systematic literature with respect to three decades: the introductory decade (1988–2000), the emerging decade (2001–2010), and the thriving decade (2011–2020).

In this study, we observed that North American researchers are more involved in Cause-Related Marketing. This may be due to the importance that CRM has had in the USA since its inception, and the acceptance that CRM has had among American companies and consumers. Such as Cone (2010) showed, 88% of the American consumers supported the cause, 85% of the consumers had a good image of the company or product supporting a noble cause and cared about it, and 90% of the consumers demanded companies to find the right cause to support. More recently, another research also studied that 87% of American consumers would purchase a CRM product if the company supported the charitable cause (business2community, 2020).

The graphical presentation of the Time-Wise Development (see Fig. 1) shows 13 articles published until 2000 (first decade), 74 articles from 2001 to 2010 (second decade), and 257 articles from 2011 to 2020 (third decade). Natarajan et al. (2016, p. 248) and Thomas et al. (2020, p. 5) verified almost similar findings of the time-wise development from 1988 to 2016. But after that, the research on Cause-Related Marketing has abruptly increased in the last two years (2019–2020). We noticed that, in 2020, the researchers are more actively involved in the CRM field than the previous years to publish the research articles.

As observed in Fig. 8, the academicians and researchers from 37 different countries have significantly contributed to CRM studies. A large portion of CRM studies are conducted in two regions (i.e., North America and Europe). Thomas et al. (2020) shows similar results. Asian (i.e., Indian and Taiwanese) researchers have taken more interest in CRM and they have been publishing more and more articles since the third decade. On the other hand, we also noticed that the CRM topic was first introduced in Western societies (with Christian tradition). But after the first decade and during the second decade, CRM studies were also introduced in Muslim countries, such as Pakistan (1 research article) and Oman (1 research article). From the third decade, the researchers also explored other Muslim countries, such as Malaysia (5 research articles), Iran (4 research articles), Egypt (4 research articles), Turkey (3 research articles), Pakistan (3 research articles), Jordan (1 research article), Indonesia (1 research article), and Bangladesh (1 research article). So, in summary, we can highlight that researchers have been exploring the Muslim world in the field of CRM after the second decade.

And about the methodological development in CRM literature (see Fig. 9), the researchers have used more quantitative studies, compared to qualitative studies. Thomas et al. (2020, p. 7) also found a similar result. Thus, the trend seems to be for quantitative studies to continue to predominate over qualitative ones in the coming years, although mixed methods are experiencing slight growth. However, the combination of both types of studies, qualitative and quantitative, could offer more complete studies on CRM.

Lastly, Table 6 presents the journals involvement to publish CRM research articles. In our study, the key publications journals are *International Marketing Review,* and *International Review on Public and Nonprofit Marketing.* Our results have been slightly different from Thomas et al. (2020)‘s and Natarajan et al. (2016)‘s. These researchers found that the *Journal of Nonprofit & Public Sector Marketing* was the one that had published more research articles on this topic. But probably this difference is because they only considered up to 2016.

**Table 6:** The list of five most cited journals from each decade

1988–2000			2001–2010			2011–2020		
Journal Name	No. of Articles	Citation	Journal Name	No. of Articles	Citation	Journal Name	No. of Articles	Citation
Journal of marketing	1	734	Journal of consumer marketing	3	369	International marketing review	25	842
Journal of public policy & marketing	1	498	Journal of business research	3	280	Journal of business ethics	11	458
Journal of consumer marketing	1	121	Journal of retailing	1	243	International journal of non-profit and voluntary sector marketing	9	407
Journal of business ethics	1	110	Journal of non-profit and public sector marketing	11	215	International journal of advertising	8	347
Journal of the academy of marketing science	2	51	Journal of advertising	5	172	Journal of marketing	2	244

Source: Own Elaboration

Our research also discovered different results from previous studies with respect to databases, partly due to the number of databases considered and the greater number of years analyzed in our study.

## Conclusion

Cause-Related Marketing (CRM) is considered as an initiative that involves a donation to a specific cause, at a specific period of time, usually with a limited donation amount, and which depends on product sales and consumer behavior.

Therefore, the main objective of this study was to provide a comprehensive systematic review of the literature on CRM, categorizing each article by time-wise development, country-wise development, methodological development, and role of journals. Cross-cultural analysis and bibliometric analysis were also included, as a new contribution of this research, in comparison to previous studies.

The main studies have been classified in three decades, which present significant differences. In the introductory decade (1990–2000), the field of CRM was introduced with limited published articles with the role of CRM in two different regions, such as North America, and Oceania.

First three Journals such as *International Marketing Review, International Review on Public and Nonprofit Marketing,* or *Journal of Nonprofit and Public Sector Marketing,* play a starring role to publish CRM research papers. In the emerging decade (2001–2010), researchers explored more regions, such as East Asia, South Asia, and the Middle East. In this time frame, mix approach studies and cross-cultural studies were introduced for the first time in the field of CRM. And in the most thriving decade (2011–2020), scholars analyzed CRM in two more regions, such as North Africa and Sub-Saharan Africa. In this era, more scholars were interested in collaborating with other geographical regions such as North America and Europe. The number of published papers on CRM grew significantly.

However, this current study has some limitations. First, this research considered only two keywords: “Cause-Related Marketing” and “Cause Marketing”. Thus, other terms might be also considered, such as “Social Cause” or “Cause–brand alliance”. Secondly, the selection of the studies was limited only to the peer-reviewed journal research articles published in English. Maybe research articles in other languages could be also interesting. Thirdly, this current study just focuses on the full-text journal papers. Abstracts, theses, working papers, and conference proceedings were ignored. Fourthly, this study has used a limited number of databases to find the research articles: SAGE Journals, JSTOR, Emerald Insight, Springer, Wiley Online Library, Elsevier, Taylor & Francis Online, and Google Scholar. Other databases, such as EBSCO and ABI/INFORM, could have been also considered.

Anyway, we also found different gaps in CRM research, so further research could be developed in these aspects. First, most academic scholars have largely focused on the developed countries, such as the USA and the UK, and less in developing countries (especially in the first and second decades). Although studies about CRM in developing countries increased in the third decade, the gap still exists. More studies are needed about developing countries because the researchers may find different results. In addition, more studies are also required to compare developed and developing countries, because researchers could find different interesting outcomes about CRM campaigns.

Secondly, the growing popularity of the internet and social media could be more considered by the companies, which could focus on digital marketing. Therefore, consumers could be more involved in a digital CRM campaign (Handa & Gupta, 2020). Only few studies have been conducted in this area, so the gap still exists, both in developing and developed countries.

Thirdly, various studies are conducted on the cross-culture context. But more research is needed to investigate the cross-cultural context, comparing developing and developed countries, and also Western and Eastern countries. Causes and consumer preferences or attitudes could be different from one country to another country.

Fourthly, few studies have been conducted in the mix approach (including qualitative and quantitative studies). More research is required for a better understanding of the mixed methodological approach in CRM. The most common and well-known approaches to mixing methods are Triangulation Design, Embedded Design, the Explanatory Design, and the Exploratory Design. These methodologies could be discussed in CRM programs.

Fifthly, profit and non-profit organizations depend on each other in CRM campaigns. When both organizations develop CRM strategies, they can acquire risk. Few studies have been conducted on profit and non-profit organizations with CRM programs; therefore, this also needs to be discussed.

Finally, and according to Chéron et al. (2012), consumers positively view those CRM campaigns that take place for extended periods of time, and they might be disappointed with short duration campaigns. Thus, time frame of the CRM campaign can have a significant impact on the consumers’ perception. Consequently, the campaign’s time duration is another factor that is needed to be more discussed by researchers.

## Appendix 1

**Table 7:** Definitions of Cause-Related Marketing

**Year**	**Author**	**Definition**
1988	Varadarajan and Menon	The process of formulating and implementing *marketing activitie***s** that are characterized by an offer from the firm to contribute a specified amount to a designated cause when consumers engage in revenue-providing exchanges that satisfy organizational and individual objectives.
1991	Smith and Alcorn	Cause marketing may be the most creative and cost-effective product ***marketing strategy*** to evolve in years, and one that directly addresses the issue of measured financial returns.
1994	Carringer	The *joining* ***together*** of a not-for-profit charity and a commercial company in an effort to raise funds and awareness for the cause while building the sales and awareness of the for-profit partner.
1996	Hawkens and Stead	Any ***marketing activity*** undertaken by a company designed to simultaneously benefit the company and the charity, or similar cause
1997	Mullen	The process of formulating and implementing ***marketing activities*** that are characterized by contributing a specific amount to a designated non-profit effort that, in turn, causes customers to engage in revenue-providing exchanges
1997	Ptacek and Salazar	***Working togethe****r* in financial concert with a charity … to tie a company and its products to a cause
1998	File and Prince	Cause related marketing has become an established part of the ***marketing mix*** in privately held companies, confirming the adoption of this marketing innovation in a new segment of business.
1998	Webb and Mohr	Cause-related marketing (CRM) campaigns provide an excellent context for delving into consumers’ interpretation of ***promotions*** with a social dimension and exploring their behavioral responses to such corporate “do-gooding.”
1999	Pringle and Thompson	***Activity*** by which a company with image, product or service to market builds a relationship with a “cause” or a number of “causes” for mutual benefit.
1999	Adkins	A c***ommercial activity*** by which businesses and charities or causes form a partnership with each other to market an image, product or service for mutual benefit
2000	Barone, Miyazaki, and Taylor	CRM is a ***strategy*** designed to promote the achievement of marketing objectives (e.g., brand sales) via company support of social causes
2001	Samu and Wymer Jr.	Cause Related Marketing (CRM) is a ***marketing program*** that tries to improve business performance and help nonprofit causes by linking donations to the purchase of a firm’s products
2001	Pringle and Thompson	A strategic ***positioning*** and marketing tool which links a company or brand to a relevant social cause or issue, for mutual benefit.”
2003	Hajjat	Cause-related marketing (CRM) involves the integration of ***marketing activities*** of a for-profit firm with fund raising requirements of a not-for-profit organization (NPO). In its basic form, CRM campaigns try to persuade consumers to buy a certain product by promising to donate something in return to a specific cause.
2003	Nowak and Clarke	Cause-related marketing is the ***firm’s contribution*** to a designated cause being tied to customers’ participating in revenue-producing transactions with the firm
2003	Docherty and Hibbert	CRM expresses ***corporation responsiveness*** to social concerns while raising funds for a good cause
2003	Cui, Trent, Sullivan, and Matiru	A ***general alliance*** between businesses and non-profit causes that provide resources and funding to address social issues and business marketing objectives.
2004	Endacott	Cause-related marketing (CRM) is a ***marketing strategy*** adopted by businesses to link their name, brand or service with a particular “good cause” service or charitable organization
2005	Kotler and Lee	Cause-Related Marketing is a ***CSR initiative*** that makes a contribution or donates a percentage of revenues to a specific cause based on product sale or usage.
2005	Berglind and Nakata	Marketing a product, service, brand, or ***company by tying it with a social cause*** (such as breast cancer detection and treatment) is the essence of cause related marketing.
2005	Lafferty and Goldsmith	A form of ***corporate philanthropy*** based on the rationale of profit motivated giving that can be viewed as a manifestation of the alignment of corporate philanthropy and enlightened business
2006	Van den Brink, Odekerken-Schröder, and Pauwels	CRM is a specific ***marketing activity*** in which the firm promises its consumers to donate company resources to a worthy cause for each sold product or service.
2006	Fromherz	Cause Related Marketing (CRM) is a ***marketing strategy*** that links together purchases of a product or service with fundraising efforts for a worthwhile charity project or cause
2006	Gupta and Pirsch	Cause-related Marketing is a process of formulating and implementing ***marketing activities*** that are characterized by an offer from the firm to contribute a specified amount to a designated cause when customers engage in revenue providing exchanges to induce favorable responses from all company stakeholders which in turn satisfy organizational and individual objectives
2008	Larson, Flaherty, Zablah, Brown, and Wiener	Cause-related marketing as any ***marketing activities*** in which company donations to a specified cause are based upon sales of specified goods or services.
2009	Kim and Lee	Cause-related marketing (CRM) is a popular ***marketing tool*** that ties a brand or a company with a social cause.
2010	Tangari, Folse, Burton, and Kees	Cause-related marketing (CRM) is a ***promotional strategy*** that combines public relations and sponsorship strategies where a company makes a philanthropic commitment to a societal need or “cause” through a specific campaign that is promoted to and requires participation from consumers.
2012	Galan-Ladero	Cause-Related Marketing is a ***CSR activity***. It is an agreement between a company and a NPO to collaborate on a social cause and obtain, in this way, a mutual benefit. The company commitment is focused on contributing (financially or in kind) to the cause in terms of sales (the donation will depend, therefore, on consumer behavior). Normally, the campaign is conducted for a certain product, for a specific period, and with a particular NPO.
2012	Chéron, Kohlbacher, and Kusuma	The firms’ ***contribution to a specified caus******e,*** where the amount of contribution is somehow dependent on customers’ purchasing behavior that can create a win-win situation both for organization and cause as well as customer
2013	Boenigk and Schuchardt	CRM as a ***strategic partnership*** between a for profit brand and a charitable organization that produces a promotional marketing campaign, with a specific proportion of the profits earned from sales of a firm’s products or services donated to the designated charitable cause
2013	Beise-Zee	A ***promotional activity*** of an organization in which a societal or charitable cause is endorsed, commonly together with its products and services as a bundle or tie-in’
2014	Stumpf and Teufl	CRM can be defined as a strategic positioning and ***marketing tool*** which links a company or a brand to a relevant social cause or issue, for mutual benefit
2016	Bergkvist and Taylor	CRM as a form of leveraged ***marketing communication******s*** (LMC), that is, marketing communications that aim for the brand to benefit from consumers’ positive associations to another object (e.g. a cause)
2017	Thamaraiselvan, Arasu, and Inbaraj	Cause-Related Marketing (CrM) is a ***marketing strategy*** that combines philanthropic activities with structured marketing efforts in both for-profit and nonprofit organizations
2018	Jung, Naughton, Tahoun, and Wang	Cause-related marketing as ***horizontal cooperative promotion,*** meaning to precede promotion with the combination of enterprise brand and non-profit organizations
2018	Sabri	Cause-related marketing (CrM), defined as a firm’s ***communication activities*** designed to promote a consumer good or service by including an offer to contribute a specified amount to a designated nonprofit cause, has become a preponderant practice
2018	Manojkumar and Sharma	Cause related marketing strategy can be defined as a ***strategy*** where a company declares to donate a percentage of revenues to a specific cause based on the revenue occurring during announced period of support
2019	Yun, Duff, Vargas, Himelboim, and Sundaram	A ***business strategy*** in which a brand partners with a cause through various types of engagements, to address both organizations’ objectives
2020	Srivastava	Cause-related marketing is a ***marketing strategy*** wherein a product/brand/company is marketed in association with a “cause”—to change the behavior or donate a percentage of revenue for the betterment of society

## Appendix 2

**Table 8:** Role of Journal in Development of CRM literature (Journal ranking with according to ICI Journals)

**Sr**	**Journal Name**	**Journal Ranking**	**First Decade**	**Second Decade**	**Third Decade**	**Total**
1	International marketing review	Q1		1	25	26
2	International review on public and nonprofit marketing	Q2		5	15	20
3	Journal of nonprofit and public sector marketing	Q3	2	12	3	17
4	International journal of nonprofit and voluntary sector marketing	Q2	1	4	10	15
5	Journal of business ethics	Q1	1	1	11	13
6	Journal of business research	Q1		3	7	10
7	Journal of marketing communications	Q1		1	8	9
8	International journal of advertising	Q1		1	8	9
9	Journal of the academy of marketing science	Q1	2	1	5	8
10	Journal of consumer marketing	Q2	1	3	4	8
11	European journal of marketing	Q1		1	7	8
12	Sustainability (Switzerland)	Q1			8	8
13	Journal of product and brand management	Q1		2	6	8
14	Psychology & marketing	Q1		2	5	7
15	Journal of advertising	Q1		5	1	6
16	Sport marketing quarterly	Q2		1	3	4
17	Journal of promotion management	Q2		1	3	4
18	Journal of consumer psychology	Q1		1	3	4
19	Total quality management and business excellence	Q1		1	3	4
20	Marketing intelligence and planning	Q2		1	2	3
21	Journal of marketing theory and practice	Q2		1	2	3
22	Journal of marketing	Q1	1		2	3
23	Journal of brand management	Q2			3	3
24	Journal of hospitality and tourism research	Q1			3	3
25	Third world quarterly	Q1			2	2
26	Sport, business and management: an international journal	Q2			2	2
27	Social responsibility journal	Q2		1	1	2
28	Nonprofit and voluntary sector quarterly	Q1		1	1	2
29	Journal of retailing and consumer services	Q1			2	2
30	Journal of retailing	Q1	1	1		2
31	Journal of public policy & marketing	Q1	1	1		2
32	Journal of current issues and research in advertising	Q2		1	1	2
33	Journal of marketing research	Q1			2	2
34	Journal of islamic marketing	Q2			2	2
35	International journal of retail and distribution management	Q1		1	1	2
36	Journal of global scholars of marketing science	Non-indexed in the ICI Journals			2	2
37	Journal of advertising research	Q1		1	1	2
38	International journal of sports marketing and sponsorship	Q2			2	2
39	International journal of contemporary hospitality management	Q1			2	2
40	International journal of business communication	Q2			2	2
41	International journal of business and management	Q4			2	2
42	European sport management quarterly	Q1			2	2
43	European business review	Q1			2	2
44	Corporate social responsibility and environmental management	Q1		1	1	2
45	Cogent business and management	Q2			2	2
46	Clothing and textiles research journal	Q2		1	1	2
47	Business process management journal	Q1			2	2
48	African journal of business management	Q4		1	1	2
49	World review of entrepreneurship, management and sustainable development	Q3			1	1
50	World applied sciences journal	Not Yet Assigned Quartile			1	1
51	Voluntas	Q1			1	1
52	Vine journal of information and knowledge management systems	Q2			1	1
53	Strategic change-briefings in entrepreneurial finance	Non-indexed in the ICI Journals		1		1
54	Social sciences	Q2			1	1
55	Social marketing quarterly	Q3		1		1
56	Social business	Non-indexed in the ICI Journals			1	1
57	Social behavior and personality	Q3			1	1
58	Services marketing quarterly	Q3		1		1
59	Service business	Q1			1	1
60	Scientific journal of administrative development	Non-indexed in the ICI Journals		1		1
61	Responsibility and sustainability. Socioeconomic, political and legal issues,	Non-indexed in the ICI Journals			1	1
62	Research journal of recent science	Non-indexed in the ICI Journals			1	1
63	Procedia-social and behavioral sciences,	Not Yet Assigned Quartile			1	1
64	Nonprofit management and leadership	Non-indexed in the ICI Journals	1			1
65	Mediterranean journal of social sciences	Not Yet Assigned Quartile			1	1
66	Marketing letters	Q1			1	1
67	Journalism & mass communication quarterly	Q1		1		1
68	Journal of sustainable tourism	Q1			1	1
69	Journal of social marketing	Q2			1	1
70	Journal of small business strategy	Q2	1			1
71	Journal of services marketing	Q1			1	1
72	Journal of security and sustainability issues	Not Yet Assigned Quartile			1	1
73	Journal of relationship marketing	Q3			1	1
74	Journal of marketing management	Q1		1		1
75	Journal of management and organization	Q2		1		1
76	Journal of management	Q1			1	1
77	Journal of macromarketing,	Q2		1		1
78	Journal of international marketing	Q1			1	1
79	Journal of international food and agribusiness marketing	Q2			1	1
80	Journal of indian business research	Q3			1	1
81	Journal of hospitality marketing and management	Q1			1	1
82	Journal of hospitality and tourism management	Q1			1	1
83	Journal of global responsibility	Non-indexed in the ICI Journals			1	1
84	Journal of fashion marketing and management	Q1			1	1
85	Journal of education for business	Q2			1	1
86	Journal of database marketing and customer strategy management	Not Yet Assigned Quartile			1	1
87	Journal of critical reviews	Not Yet Assigned Quartile			1	1
88	Journal of consumer affairs	Q1			1	1
89	Journal of asian business strategy	Non-indexed in the ICI Journals			1	1
90	Journal of applied marketing theory	Non-indexed in the ICI Journals			1	1
91	Journal of applied business research	Q4	1			1
92	Journal of advances in business management	Non-indexed in the ICI Journals			1	1
93	International review of management and marketing,	Not Yet Assigned Quartile			1	1
94	International review of management and business research	Non-indexed in the ICI Journals			1	1
95	International journal on food system dynamics	Q2		1		1
96	International journal of social sciences & education	Non-indexed in the ICI Journals			1	1
97	International journal of scientific & technology rsearch,	Not Yet Assigned Quartile			1	1
98	International journal of research in marketing	Q1			1	1
99	international journal of management practice	Q4			1	1
100	International journal of logistics research and applications	Q1			1	1
101	International journal of emerging markets	Q2		1		1
102	international journal of electronic customer relationship management	Q3		1		1
103	International journal of economics and management	Q3			1	1
104	International journal of business forecasting and marketing intelligence	Non-indexed in the ICI Journals			1	1
105	International journal of business ethics in developing economies	Non-indexed in the ICI Journals			1	1
106	International journal of business and society	Q3			1	1
107	International business research	Non-indexed in the ICI Journals			1	1
108	Indian journal of marketing	Q3			1	1
109	Iim kozhikode society & management review	Non-indexed in the ICI Journals			1	1
110	Health marketing quarterly	Q3			1	1
111	Global sport business journal	Non-indexed in the ICI Journals			1	1
112	Global journal of finance and management	Non-indexed in the ICI Journals			1	1
113	Global business review	Q2			1	1
114	Global business and economics review,	Q4			1	1
115	Geoforum	Q1			1	1
116	Food policy	Q1			1	1
117	European journal of management and business economics	Q2			1	1
118	Euromed journal of business	Q1			1	1
119	Environment and behavior	Q1			1	1
120	Economics and sociology	Q2			1	1
121	Corporate reputation review	Q3			1	1
122	Contemporary management research	Q3		1		1
123	Consumption markets and culture	Q1			1	1
124	Computers in human behavior	Q1			1	1
125	Business horizons	Q1		1		1
126	Business ethics-a european review	Non-indexed in the ICI Journals			1	1
127	Benchmarking	Q2			1	1
128	Bar-brazilian administration review	Q3		1		1
129	Baltic journal of management	Q2		1		1
130	Australian journal of management	Q2			1	1
131	Australasian marketing journal	Q2			1	1
132	Asia-pacific social science review	Q2			1	1
133	Asian journal of social sciences & humanities	Non-indexed in the ICI Journals			1	1
134	Asian journal of communication	Q1			1	1
135	Asian journal of business research	Q3			1	1
136	Asia pacific journal of research	Non-indexed in the ICI Journals			1	1
137	Asia pacific journal of marketing and logistics	Q2			1	1
138	Asia pacific journal of marketing & management review	Non-indexed in the ICI Journals			1	1
139	An international research review	Non-indexed in the ICI Journals		1		1
140	Ams review	Non-indexed in the ICI Journals			1	1
141	Agribusiness	Q2			1	1

## Footnotes

1 Except in 2009, the only year with a slight decrease compared to the previous year (− 0.3%) but recovered in the following year.

2 In 2000, Enron was the seventh-largest corporation by growth revenue in the gas and utility market in the United States territory. At the end of 2001, Enron started to face financial strategy manipulation. Consequently, on 2nd December 2001, Enron declared bankruptcy because of the fraudulent activity of corporate governance.

3 The ISO standard also consists of the ISO 14001 (Environmental Management System) and ISO 9001 (Quality Management System).

4 Carroll (1979) presented the “Carroll’s CSR Pyramid Theory”, which consisted of the economic, legal, ethical, and philanthropic lines.

5 Elkington (1994) presented the “Triple Bottom Line Theory”, which explained the economic, social, and environmental lines.

6 Freeman (1984) introduced the “Stakeholder Theory”, which described the role and participation of stakeholders to enchance CSR.

7 Matten and Crane (2005) described “the role of the corporation in administering citizenship rights for individuals”.
